# Reply to Canceill et al. Analyzing the Clinical Potential of Cold Atmospheric Plasma in Dentistry as an Alternative to Antibiotic Therapy. Comment on “Gross et al. Guided Plasma Application in Dentistry—An Alternative to Antibiotic Therapy. *Antibiotics* 2024, *13*, 735”

**DOI:** 10.3390/antibiotics14030327

**Published:** 2025-03-20

**Authors:** Tara Gross, Loic Alain Ledernez, Laurent Birrer, Michael Eckhard Bergmann, Markus Jörg Altenburger

**Affiliations:** 1 Department of Operative Dentistry and Periodontology, Center for Dental Medicine, Medical Center–University of Freiburg, Faculty of Medicine, Albert-Ludwigs-University of Freiburg, Hugstetter Straße 55, 79106 Freiburg, Germany; tara.gross@uniklinik-freiburg.de (T.G.);; 2 Center for Tissue Replacement, Regeneration & Neogenesis (GERN), Department of Operative Dentistry and Periodontology, Medical Center, Faculty of Medicine, University of Freiburg, 79108 Freiburg, Germany; 3 Laboratory for Biomedical Microtechnology, Department of Microsystems Engineering (IMTEK), University of Freiburg, 79110 Freiburg, Germany; ledernez@imtek.de (L.A.L.); bergmann@imtek.de (M.E.B.); 4 Department of Orthodontics, Center for Dental Medicine, Medical Center–University of Freiburg, Faculty of Medicine, Albert-Ludwigs-University of Freiburg, Hugstetter Straße 55, 79106 Freiburg, Germany

Thank you for your interest and your thoughtful comment on our paper “Guided Plasma Application in Dentistry—An Alternative to Antibiotic Therapy”. As this is one of the initial investigations into this approach, we recognize that further studies are necessary to fully explore its potential. However, as with any new field, it is important to start somewhere, and these preliminary findings serve as a foundation for future research.

We hope that the authors agree that the biofilm on the implant has a pathological effect on the health of peri-implant tissues. This is why antibiotics come into play, locally administered or given systemically, as stated by the authors. The systemic administration of antibiotics shows the great disadvantage of the therapy. We fight a local problem with systemic treatment given all its side effects and adverse effects. To disintegrate the biofilm and to kill bacteria locally should be the goal of future biofilm management. Following this idea, the device was developed to precisely guide and apply a plasma directly onto the implant surface. We have chosen this experimental setup to investigate whether this innovative technology holds its promise to disrupt the biofilm and eradicate bacteria locally, on the implant surface.

It is undeniable that antibiotic resistance is a global issue extending beyond the field of dentistry. As mentioned in our manuscript, there is an excessive use of antibiotics, particularly in dentistry, and the development of resistance can have fatal consequences [1].

Your commentary mentions that there is no advantage in relation to antibiotics as the resistance levels for penicillin and aminopenicillin are as low as 3% [2]. We recommend taking further literature into consideration such as, for example, Karbach et al. (2007), who found that up to 37% of isolated mixed cultures sampled from periimplantitis sites were non-susceptible to tested antibiotics agents [3]. These findings were lower when the pathogens were tested separately, which might explain the findings generated by Meinen [4]. Moreover, we shall take into consideration that antibiotic consumption is increasing in all countries (from middle-income to high-income countries). Thus, more than one country should be taken into consideration.

However, your dismissal of CAP as a viable alternative to traditional antibiotics overlooks its potential as a promising adjunct to current treatment approaches. We are not suggesting that CAP is a panacea for all antibiotic resistance issues but rather that it could be a valuable addition to existing therapies.

We never claimed that these results could be directly translated into clinical practice without further investigation. Rather, in the Discussion Section, we emphasized the need to validate these promising findings in future studies, particularly by expanding the bacterial strains tested and addressing the complexities of the biofilms found in real-world clinical conditions. This research is an essential first step in advancing the technology and evaluating its clinical applicability.

Your objection to the experimental model of applying CAP directly to the implant site implies that you may misunderstand the purpose of our study. We are fully aware that clinical practices must account for additional factors such as gingival tissue health. The experimental approach chosen was a preliminary one, and our study was meant to lay the groundwork for future research that will explore the broader implications of CAP in clinical practice. We appreciate your perspective, but we would like to clarify that our study is not an endpoint but rather a beginning of a larger research effort.

We will definitely take your critique into account as we proceed with additional studies.
